# Electrochemotherapy with Bleomycin Enhances Radiosensitivity of Uveal Melanomas: First In Vitro Results in 3D Cultures of Primary Uveal Melanoma Cell Lines

**DOI:** 10.3390/cancers13123086

**Published:** 2021-06-21

**Authors:** Miltiadis Fiorentzis, Ekaterina A. Sokolenko, Nikolaos E. Bechrakis, Saskia Ting, Kurt W. Schmid, Ali Sak, Martin Stuschke, Berthold Seitz, Utta Berchner-Pfannschmidt

**Affiliations:** 1 Department of Ophthalmology, University Hospital Essen, University of Duisburg-Essen, Hufeland Str. 55, 45147 Essen, Germany; ekaterina.sokolenko@uk-essen.de (E.A.S.); nikolaos.bechrakis@uk-essen.de (N.E.B.); utta.berchner-pfannschmidt@uk-essen.de (U.B.-P.); 2 Institute of Pathology, University Hospital Essen, University of Duisburg-Essen, Hufeland Str. 55, 45147 Essen, Germany; saskia.ting@uk-essen.de (S.T.); kw.schmidt@uk-essen.de (K.W.S.); 3 Department of Radiotherapy, West German Cancer Center, University Hospital Essen, University of Duisburg-Essen, 45147 Essen, Germany; ali.sak@uk-essen.de (A.S.); martin.stuschke@uk-essen.de (M.S.); 4 Department of Ophthalmology, Saarland University Medical Center, Kirrberger Str. 100, 66421 Homburg, Germany; berthold.seitz@uks.eu

**Keywords:** radiation therapy, electrochemotherapy, bleomycin, uveal melanoma, 3D tumor spheroids, cytotoxic effects, long-time survival

## Abstract

**Simple Summary:**

Uveal melanoma (UM) is the most common primary intraocular tumor in adults. Treatment options for UM include radiotherapy, thermotherapy and tumor resection. Electrochemotherapy (ECT) is a new therapeutic modality for local tumor control in various cancer entities. The current study assesses the radiosensitizing effect of concomitant ECT with bleomycin and irradiation on 3D tumor spheroids with primary and radioresistant uveal melanoma cell lines. The evaluation of the radiosensitizing effect of ECT as a drug delivery system was based on the changes in the spheroid growth, the cell viability as well as the cytotoxic long-term effect of the combined treatment. The primary cell lines showed a higher radiosensitivity and required lower irradiation and bleomycin doses in comparison to cell lines originating from previously irratiated tumors. ECT should be further assessed for its applicability in clinical settings as a therapeutic radiosensitizing option for radioresistant tumors.

**Abstract:**

Electrochemotherapy (ECT) is emerging as a complementary treatment modality for local tumor control in various cancer entities. Irradiation is an established therapeutic option for oncologic patients, which is commonly combined with chemotherapy due to its insufficient targeting ability. The efficiency of radiotherapy for tumors can be enhanced with different radiosensitizers. ECT can potentiate the radiosensitizing effect of chemotherapeutic agents such as bleomycin. The present study aims to evaluate the radiosensitizing effect of concomitant ECT with bleomycin on 3D tumor spheroids with primary and radioresistant uveal melanoma cell lines (UPMD2, UPMM3, UM92.1, Mel270) and irradiation. The changes in the spheroid growth and the cell viability as well the cytotoxic long-term effect of the combination treatment were evaluated with various combinations of electroporation settings and bleomycin concentrations as well as radiotherapy doses. A broad range of radiosensitivity was documented among the spheroids from different uveal melanoma cell lines. The primary cell lines showed a higher radiosensitivity and required lower irradiation and bleomycin doses. The maximal tumor control with a reduction of cell survival <10% was achieved with a 5 Gy irradiation only in the primary uveal melanoma cell lines and in combination with all tested ECT settings, whereas the same result could be obtained in UM92.1 spheroids only after ECT with 20 Gy irradiation. Based on the spheroid growth and the measurement of the cross-sectional area, the Mel270 spheroids, originating from a previously irradiated recurrent uveal melanoma, required higher doses of bleomycin and ECT settings after irradiation with 5 Gy in order to achieve a significant growth reduction. No significant difference could be demonstrated for the reduction of cell viability in the combination therapy with 20 Gy and 1000 V/cm between 1 and 2.5 µg/mL bleomycin even in Mel270 spheroids, underlying the importance of a drug delivery system to potentiate the radiosensitizing effect of agents in lower doses. ECT should be further assessed for its applicability in clinical settings as a therapeutic radiosensitizing option for radioresistant tumors and a sufficient local tumor control with lower chemotherapy and irradiation doses.

## 1. Introduction

Uveal melanoma (UM) is the most common primary intraocular tumor in adults and has an incidence of approximately 5.1 per million per year in the USA and of 1.3–8.6 per million per year in Europe, following a north-to-south decreasing gradient from a maximum of 8 per million per year in Denmark and Norway to a minimum of 2 per million per year in the Mediterranean region [1,2]. Other predisposing factors are a preexisting nevus, melanocytic lesions, a fair complexion, a light iris and *BRCA1*-associated protein (*BAP1)* tumor predisposition syndrome [3]. A UM arises from neoplastic melanocytes along the uveal tract. The most common site is the choroid (85%) with the remainder arising in the ciliary body or the iris [1,2,3,4,5]. The majority of UMs are located in the globe at first presentation; however, large lesions may show an extraocular extension along the optic nerve or the vortex veins [3].

A UM has distinct molecular features compared with other melanoma subtypes. The cutaneous melanoma-associated mutations such as v-raf murine sarcoma viral oncogene homolog B1 (*BRAF*), neuroblastoma RAS viral oncogene homolog (*NRAS*) and neurofibromatosis type 1 (*NF1*) are not common in a UM, which are characterized by different oncogenic genes or mutations with a loss of function [6]. By next-generation sequencing efforts on UM tumors, several driver genes have been detected. The most frequent among these are mutations of guanine nucleotide-binding protein Q polypeptide/guanine nucleotide-binding protein alpha-11 (*GNAQ/GNA11*), which appear in a mutually exclusive manner and occur in approximately 90% of posterior UMs [7]. Other mutations such as BAP1, splicing factor 3B subunit 1 (*SF3B1*), eukaryotic translation initiation factor 1A, X-linked (*EIF1AX*) and telomerase reverse transcriptase promoter (*TERTp*) have also been associated with UMs. A direct lineage between cytogenetic alterations and prognosis has been described and M3, 8q+ 6p+ and 1p- can consequently be used as biomarkers of prognostic importance [6,7].

The primary treatment options for a UM include radiotherapy (brachytherapy or teletherapy), transpupillary thermotherapy and tumor resection (transscleral resection, endoresection, enucleation) [1,2]. The ocular treatment is selected according to the size and location of the tumor individually for each patient. The aim of therapy is to conserve the eye with useful vision as well as to minimize the risk of metastasis [3,5]. A disseminated UM can be treated with adjuvant immunotherapy, chemotherapy and molecular targeted therapy [8].

Electrochemotherapy (ECT) is a nonthermal treatment modality that combines the application of electric pulses to the tumor tissue with chemotherapeutic agents [9,10]. ECT enhances the permeability of the cell membrane and therefore the cytotoxicity of otherwise impermeant or poorly permeant anticancer drugs such as bleomycin and cisplatin through reversible poration [11]. In addition to increasing the drug uptake in the tumor cells, ECT can generate reactive oxygen species [12]. According to the ESOPE protocol, ECT involves the application of eight 100 µs pulses at a 1 or 5000 Hz frequency and specified electric field (V/cm) [13]. The application of short and intense electrical pulses increases the efficacy of chemotherapeutic drugs [9,10,13,14,15]. There is little knowledge about the effect of ECT in UMs [16,17].

Moreover, ECT has been reported as a radiosensitizing agent for radiation therapy, postulating that a single session before irradiation can significantly enhance the tumor response. The combination of electrochemotherapy preceding irradiation leads to an increased radioresponse with an enhancement factor of up to 4.6 using a radiomimetic (bleomycin) or a radiosensitizing (cisplatin) drug [12,18,19,20]. The improved antitumor effectiveness is attributed to the increased drug accumulation in the tumors due to electroporation phenomenon, secondly to the generation of reactive oxygen species by electric pulses and lastly to the vascular alteration, particularly the antivascular effects [12]. The radiosensitizing effect of ECT and other drug delivery systems has already been documented for intestinal colon cancer, sarcomas, radioresistant adenocarcinomas, fibrosarcomas and mammary carcinomas in vitro und in vivo [18,19,21,22]. Concurrent ECT and brachytherapy showed a radiosensitizing effect with an increased local tumor control in patients with inoperable endometrial cancer as well as cutaneous spinocellular carcinomas [23].

Our study examines the radiosensitizing effect of ECT with various drug concentrations as well as alternating pulse and radiation settings in 3D spheroids using two established UM cell lines (UM92.1, Mel 270) and two primary UM cell lines (UPMD2, UPMM3). Not only the spheroid growth but also the cell viability, cell survival and the long-term cytotoxic effect of the tumor spheroids were assessed in order to determine the efficacy of concurrent irradiation and ECT in a primary UM.

## 2. Results

### 2.1. Characterization of 3D Tumor Spheroids of UM Cell Lines and UPM Cells

**Figure 1:**
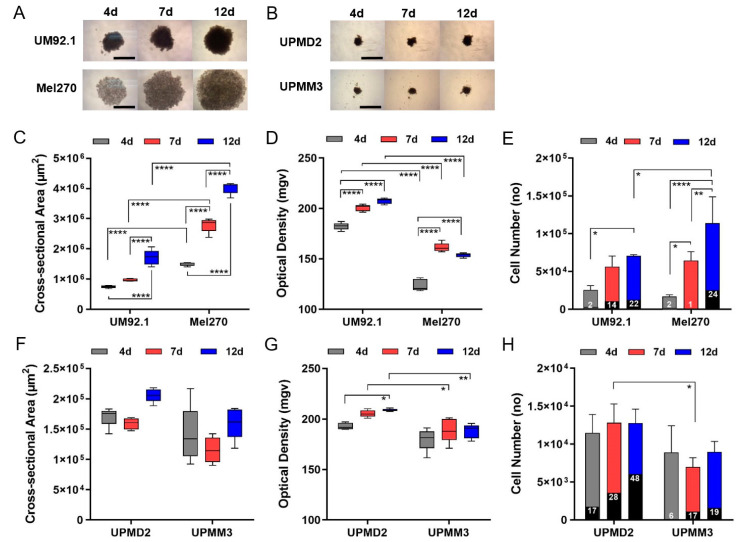
Generation of 3D tumor spheroids from UM and UPM cell lines. UM or UPM cells were seeded at 5000 cells per well. (**A**) Spheroids generated from UM cell lines UM92.1 and Mel270. Representative images are shown at day 4, 7 and 12 (4× magnification; scale bar of (**A**) represents 1000 µm). (**B**) Spheroids generated from UPM cell lines UPMD2 and UPMM3. Representative images are shown at day 4, 7 and 12 (4 × magnification; scale bar of (**B**) represents 1000 µm). (**C**,**F**) Spheroid cross-sectional area (µm^2^) and (**D**,**G**) optical density (mgv, mean grey value) were determined (*n* = 5 each time point). (**E**) UM spheroids or (**H**) UPM spheroids were trypsinized, stained with trypan blue and live/dead cell numbers were counted at day 4, 7 and 12. Dead cells are indicated by black columns; % of dead cells is given. A statistical analysis was performed using a two-way ANOVA and Tukey’s multiple comparisons test; significance levels are indicated * *p* < 0.05, ** *p* < 0.01, **** *p* < 0.001.

Spheroids were generated from two UM cell lines, UM92.1 or Mel270, and from two uveal primary melanoma (UPM) cells lines, UPMD2 or UPMM3. The characteristics of the cell lines are summarized in Table 1. During a 4- to 12-day time of culturing, all cell lines produced uniform-sized spheroids with a specific size and appearance of cell line (Figure 1). UM92.1 cells produced spheroids with an increasing size and compactness while Mel270 cells aggregated into large flat spheroids with an increasing size but low density/compactness when compared with UM92.1 (Figure 1A,C,D). Spheroid live/dead cell numbers increased until day 12 (Figure 1E). In contrast, the UPM cell lines produced much smaller spheroids with a constant size and compactness (Figure 1B,F,G). The small sizes of the UPM spheroids were reflected by constant low cell numbers. However, UPMD2 spheroids contained increasingly high numbers of dead cells when compared with other spheroid types (Figure 1H).

### 2.2. Impact of Combination Treatment on 3D Tumor Spheroid Growth

**Figure 2:**
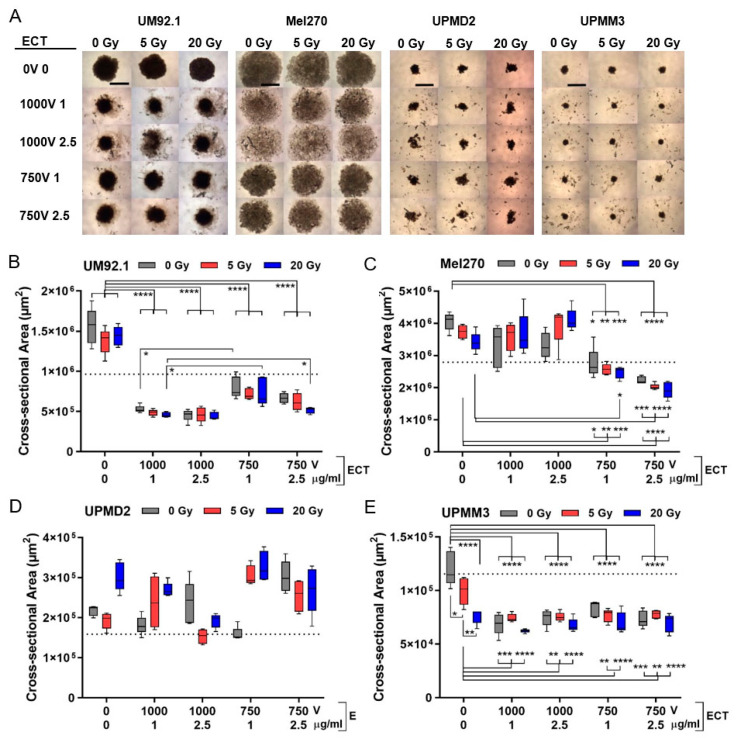
3D tumor spheroid growth after combination treatment. At day 4, spheroids were either irradiated (dose 5 or 20 Gy) or treated with ECT (electroporated 1000 V or 750 V in the presence of 1 µg/mL or 2.5 µg/mL bleomycin) or spheroids were irradiated followed by ECT. Five days after treatment (at day 12) the effect on spheroid growth was determined. (**A**) Representative spheroids are shown (4 × magnification; scale bar of (**A**) represents 1000 µm). (**B**–**E**) Corresponding cross-sectional area of spheroids (*n* = 5 each condition). Additionally, the mean cross-sectional area of spheroids before treatment at day 7 is shown (dotted line; UM92.1: 9.7 × 10^5^ µm^2^; Mel270: 2.8 × 10^6^ µm^2^; UPMD2: 1.6 × 10^5^ µm^2^; UPMM3: 1.2 × 10^5^ µm^2^). A statistical analysis was performed using a two-way ANOVA and Tukey’s multiple comparisons test; * *p* < 0.05, ** *p* < 0.01, *** *p* < 0.005, **** *p* < 0.001.

The different tumor spheroids may reflect heterogeneous uveal melanomas and were therefore subjected to radiation and/or ECT on day 7. The short-time cytotoxic effect on the spheroid growth of different UM and UPM spheroids was observed five days after treatment (at day 12). Overall, radiation had little effect on spheroid morphology whereas the ECT dose dependently changed the spheroid size and appearance in a cell line-specific manner (Figure 2). In terms of UM92.1 and Mel270, ECT led to a spheroid size reduction when compared with untreated or radiation only (Figure 2A–C). Herein, ECT with a 1000 V strength reduced the size of UM92.1 spheroids more efficiently than ECT with 750 V while Mel270 spheroids disintegrated in enlarged loose cell aggregates (Figure 2A–C). However, UPM spheroids were differently affected by the treatments (Figure 2A,D,E). ECT led small fragments to separate off the spheroids and caused irregular formed fragments with a highly variable size of UPMD2 spheroids (Figure 2A,D). In contrast, either a 20 Gy radiation or ECT at any strength significantly reduced the size of UPMM3 spheroids when compared with untreated or 5 Gy radiation (Figure 2E).

Overall, ECT with both bleomycin dosages of 1 or 2.5 µg/mL were effective in the size reduction of spheroids and radiation prior to ECT had little additional effects on the spheroid size. Strikingly, the treatments led to a reduction in the spheroid size not only compared with untreated spheroids at day 12 but also compared with untreated spheroids at day 7 indicating the shrinking of the spheroids below the initial size (Figure 2B,C,E; dotted line). Several mechanisms such as the destruction of morphology, growth inhibition and/or apoptotic/necrotic processes may have contributed to spheroid shrinking.

### 2.3. Proliferation and Necrosis of 3D Tumor Spheroids in Response to Combination Treatment

**Figure 3:**
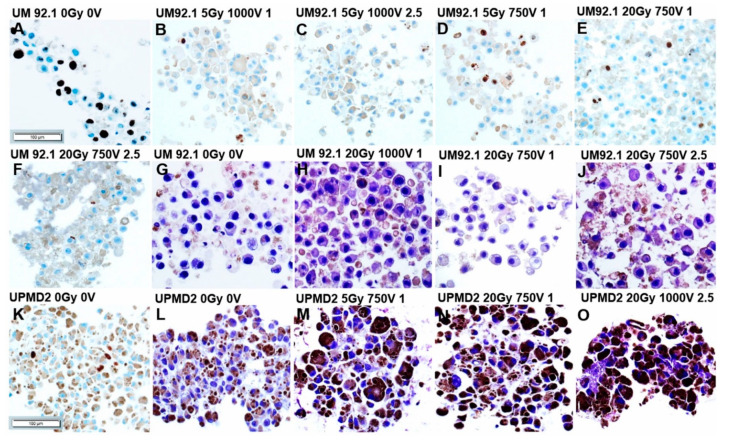
Immunohistochemistry of 3D tumor spheroids treated with combination therapy. Tumor spheroids were irradiated (dose 5 or 20 Gy) and treated with ECT (electroporated 1000 V or 750 V in the presence of 1µg/mL or 2.5 µg/mL bleomycin) or were left untreated (0 Gy 0 V). (**A**–**F**,**K**) UM92.1 or UPMD2 spheroids (*n* = 3–6 each condition) were stained for proliferation marker Ki67; (**G**–**J**,**L**–**O**) and/or stained with hematoxylin. Representative images are shown (200 × magnification; scale bar of Figure 3 represents 100 µm).

The detection of Ki67 in sections of UM92.1 spheroids revealed that the cell proliferation was strongly reduced with ECT 1000 V compared with 750 V either with 5 or 20 Gy (Figure 3A–F). Additionally, hematoxylin staining of UM92.1 spheroids revealed that bleomycin dose-dependently increased the regressive processes with pathological changes indicating apoptosis and/or necrosis (Figure 3G–J). The UPMD2 spheroid cells were massively pigmented and only a few cells stained Ki67 positive indicating a low proliferation rate (Figure 3K). Furthermore, regressive cellular changes were enhanced in the UPMD2 spheroid cells already in response to a low dose combination treatment (Figure 3L–O).

### 2.4. Viability of 3D Tumor Spheroids in Response to Combination Treatment

**Figure 4:**
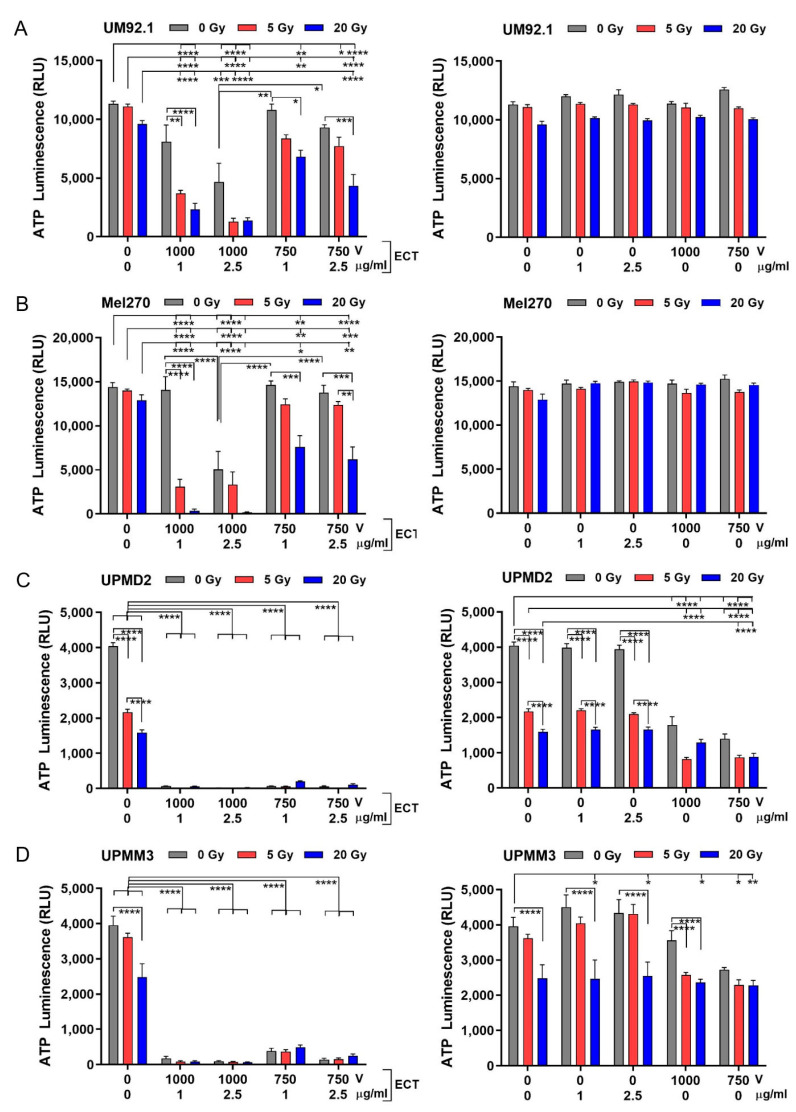
Cytotoxic effects of radiation and ECT on 3D spheroid viability. (**A**) UM92.1 spheroids, (**B**) Mel270 spheroids, (**C**) UPMD2 spheroids and (**D**) UPMM3 spheroids were irradiated (dose 5 or 20 Gy) or treated with ECT (electroporated 1000 V or 750 V in the presence of 1 µg/mL or 2.5 µg/mL bleomycin) or spheroids were irradiated followed by ECT (left side of the graphs). In addition, tumor spheroids were treated with bleomycin (1 µg/mL or 2.5 µg/mL) or electroporation (1000 V or 750 V) alone (right side of the graphs). Cytotoxic effects on spheroid viability (*n* = 5–8) were assessed by measuring the ATP content of lysed spheroid cells using a 3D cell viability assay (ATP Luminescence, RLU Relative Light Units). Data are means ± SD. A statistical analysis was performed using a two-way ANOVA and Tukey’s multiple comparisons test; * *p* < 0.05, ** *p* < 0.01, *** *p* < 0.005, **** *p* < 0.001.

In order to evaluate the cytotoxic mid-term effects of the treatments, the spheroid cell viability was analyzed after another week in the culture (12 days after treatment). The cell viability of UM spheroids or UPM spheroids were differently affected by the treatments (Figure 4). UM spheroid viability was only marginally affected by irradiation whereas ECT significantly reduced the spheroid viability at 1000 V strength with 2.5 µg/mL bleomycin (Figure 4A,B; left graphs). However, the combination treatment was more efficient in the reduction of viability than either treatment alone. The most efficient combination to reduce the viability of the UM spheroids was ECT 1000 V strength with 1 or 2.5 µg/mL bleomycin together with 5 or 20 Gy radiation (Figure 4A,B; left graphs). Neither bleomycin alone nor electroporation only had any effect on the spheroid cell viability (Figure 4A,B; right graphs).

In contrast, the UPM spheroid cell viability was reduced already by radiation. Strikingly, ECT on its own as well as in combination with radiation dramatically reduced the spheroid cell viability (Figure 4C,D; left graphs). Bleomycin alone had no effect on the UPM spheroid cell variability. However, electroporation alone or additionally to radiation was able to reduce the viability significantly (Figure 4C,D; right graphs). The different spheroids displayed a broad range of radiosensitivity and/or electro/chemosensitivity, with UPM spheroids much more sensitive to radiation and/or ECT than UM spheroids.

### 2.5. Cytotoxic Long-Term Effects on Tumor Spheroid Cells after Combination Treatment

**Figure 5:**
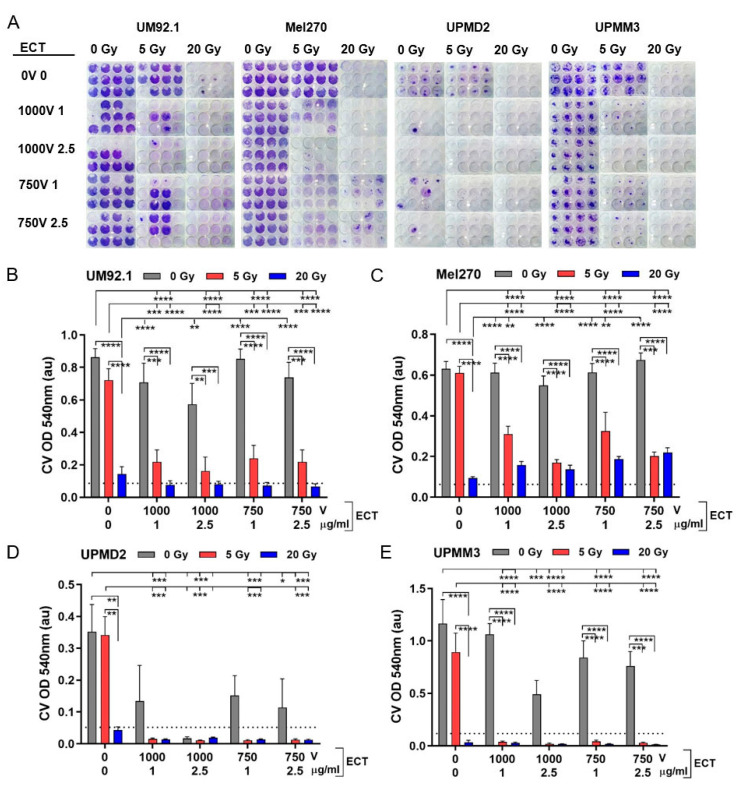
Long-term cytotoxicity of the treatments on tumor spheroid cells. At day 7, tumor spheroids were either irradiated (single dose 5 or 20 Gy) or treated with ECT (electroporation at 1000 V or 750 V in the presence of 1 µg/mL or 2.5 µg/mL bleomycin) or spheroids were first irradiated followed by ECT. The long-term cytotoxic effects on spheroids were determined in a spheroid cell survival assay. Each individual treated spheroid was allowed to attach to a flat bottom well at day 12. Spheroids were maintained in a culture to enable the out-growth of living cells. After eight weeks in the culture, the surviving cells were stained with Crystal Violet (CV). (**A**) Representative images of CV staining each condition are shown. (**B**–**E**) Measurements of CV staining (OD 540 nm, au: absorbance units, *n* = 12 each condition). Additionally, the 10% level of the mean value of untreated spheroids (0 Gy 0 V 0 µg/mL bleomycin) is shown in order to indicate the maximal tumor control potential (dotted line). Data are means ± SD. A statistical analysis was performed using a two-way ANOVA and Tukey’s multiple comparisons test; * *p* < 0.05, ** *p* < 0.01, *** *p* < 0.005, **** *p* < 0.001.

In order to determine the tumor control potential of the treatments, we analyzed the cytotoxic long-term effects of the treatments in a spheroid cell survival assay after another eight weeks in the cell culture (61 days after treatment). Basically, UM or UPM spheroids displayed a different ability of cell out-growth and proliferation in a long-time culture. The untreated UM spheroid cells formed a confluent and homogeneous cell monolayer (UM92.1, Mel270: 100% cell density) while UPM spheroid cells remained as a subconfluent and inhomogeneous monolayer (UPMD2: 60–70%; UPMM3: 80–90% cell density). The respective cell density was reflected by Crystal Violet (CV) staining of the cell nuclei/DNA (Figure 5A; representative images of the cell cultures received from untreated spheroids (0 Gy, 0 V)).

The different spheroid types exhibited different long-term outcomes in response to the treatments. A 5 Gy radiation of UM or UPM spheroids had only marginal cytotoxic effects on cells resulting in a nearly confluent monolayer while 20 Gy radiation reduced the cell survival close to <10% of the untreated, which we defined as the maximum tumor control potential (Figure 5B–E; dotted line). ECT alone did not inhibit the cell survival of UM spheroids (Figure 5B,C). In contrast, ECT dose-dependently reduced the cell survival of UPM spheroids (Figure 5D,E). In the case of UPMD2 spheroids, ECT of either dose reduced the cell survival below 50% compared with the untreated. Moreover, ECT 1000 V with 2.5 µg/mL bleomycin greatly reduced the UPMD2 cell survival to <10% of the untreated spheroids reaching the maximal tumor control potential (Figure 5D; dotted line). Concerning UPMM3 spheroids, ECT 1000 V, 2.5 mg/mL bleomycin reduced the cell survival of UPMM3 spheroids below 50% of the untreated (Figure 5E).

Furthermore, radiation with 5 Gy in combination with ECT significantly reduced the cell survival of UM spheroids below 50% and conferred the maximum tumor control potential of all UPM spheroids (Figure 5B–E). Likewise, a maximum tumor control potential was obtained with 20 Gy radiation alone or additional to ECT (Figure 5B–E, dotted line).

Taken together, the long-term survival assay of treated spheroids revealed that ECT combined with radiation could increase the cytotoxic effects of either treatment alone and conveyed the maximal tumor control potential on all spheroid types investigated.

## 3. Discussion

This study demonstrated the increased effect and delivery of radiosensitizing agents after concomitant radiotherapy and ECT in uveal melanoma 3D cell cultures. ECT enhanced the tumor response by reducing the cell viability and increasing the long-term cytotoxic effect in primary uveal cell lines. The variable response to the treatment among the tested cell lines could be attributed to differences in the intrinsic tumor cell responsiveness and sensitivity to the drug used in conjunction with electric pulses or/and radiation [20]. The radiosensitizing effect of chemotherapeutic agents such as bleomycin has already been shown in many in vitro and in vivo studies and clinical trials [30,31,32,33].

When bleomycin was administered in combination concurrently with fractionated radiation, the locoregional control and survival in patients was improved by up to 30% [33,34,35].

The discussed underlying mechanism was not only the increased delivery of the drug to the tumors by electroporation but also the ability of bleomycin to directly bind to DNA, resulting in the reduced synthesis of DNA, RNA and proteins, therefore inducing single- and double-strand DNA breaks and cell death. The effect was dose-dependent. The cytotoxic effect of bleomycin was also potentiated by chemicals that produce superoxides as did X-rays [36,37]. It has been speculated that the DNA damage caused by the increased drug concentration sensitizes the cells to subsequent irradiation. In a study, the potentiation factor for the tumor radiation response for single-dose irradiation was measured at 1.9 for ECT with bleomycin and 1.6 with cisplatin [19,38]. Various studies have reported the beneficial practice of concomitant ECT and irradiation where others have discussed the conduction of ECT prior to irradiation [19,20,21,22]. Another study demonstrated that the increased radiation response of tumors that were exposed to electric pulses was ascribed to radiobiologically-relevant tumor hypoxia and to the induction of reactive oxygen species, effects that counteract each other. Prolonging the time between bleomycin administration and irradiation could restitute the tumor oxygenation before the tumor irradiation [19]. Subsequently, the effect of the interval between bleomycin-ECT and irradiation on reactive oxygen species as well as their role in tumors after ECT has not yet been adequately addressed.

A good potentiation of the radiation response without drug delivery systems such as electroporation was only postulated in an in vitro study with colon tumor cells and with high doses of the applied chemotherapeutic agents [39]. In vivo studies on mice demonstrated the potentiation of radiation with high doses of bleomycin either after a single irradiation dose or in a fractionated regime up to 1.23-fold [22,31,32]. Drug delivery systems such as the incorporation of the drug into liposomes or other vehicles and local drug administration can be used to increase the drug delivery to tumors [40]. Electroporation as a drug delivery system can effectively increase the potentiation of bleomycin and cisplatin cytotoxicity and radiosensitivity [14]. In vivo ECT of tumors in experimental as well as clinical settings is feasible and effective for local tumor control and requires low drug doses that otherwise have minimal or no antitumor effectiveness without the subsequent application of electric pulses to the tumors. The drug accumulation increases 2-fold for cisplatin and 4-fold for bleomycin [13,14].

The combination of ECT with cisplatin and irradiation has already been examined in cell lines and tumors in mice and a patient. The study showed a potentiation of the radiation response by 1.4 after ECT with cisplatin and concurrent irradiation compared with tumors that were treated with a combined cisplatin and irradiation treatment and 1.6 compared with tumors that were irradiated only. The suggested mechanism was the increased cisplatin accumulation in the cells of tumors [18]. Another study examined and proved that the potentiation of the radiation response after ECT with bleomycin was 1.9-fold compared with tumors that were irradiated only and those that were concomitantly treated with bleomycin. The effect of bleomycin and irradiation alone without a drug delivery system did not lead to an increased potentiation as the low dose that used bleomycin alone had no radiosensitizing effect [19].

In our study, the maximal tumor control with a reduction of cell survival to <10% was achieved with a 5 Gy irradiation only in the primary uveal melanoma cell lines and in combination with all tested ECT settings. In the UM92.1 cell line, the same effect was obtained only after irradiation with 20 Gy for all ECT parameters. In the Mel270 cells, the maximal tumor control could not be achieved either with ECT alone, irradiation alone or with any tested combination treatment. This could be attributed to the origin of the Mel270 cells, which arose from a large recurrent tumor after prior irradiation [25]. The UM92.1 cells were obtained from a massive tumor mass that had destroyed the eye and orbit and led to metastases although this tumor had a disomy of chromosome 3 and expressed BAP1. An EIF1AX mutation was detected, which has been associated with the development of metastases in disomy 3 uveal melanomas [27]. These characteristics could explain the radioresistance to the tested settings for the Mel270 cell line as well as the higher dose irradiation and ECT settings needed for the treatment of the UM92.1 cell line. Interestingly, the spheroid growth based on the cross-sectional area showed a significant response in Mel270 after a combined treatment with 5 Gy and 750 V and 1 or 2.5 µg/mL bleomycin in comparison with the combination with 1000 V. This effect could be explained through the wide dispersity of the cells after the electric pulse distribution, leading to optically larger spheroids and a greater diameter despite the significant reduction of the cell viability and the central necrosis. No significant difference could be demonstrated for the reduction of the cell viability in the combination therapy with 20 Gy and 1000 V between 1 and 2.5 µg/mL bleomycin even in the radioresistant Mel270 spheroids, underlying the importance of a drug delivery system to potentiate the radiosensitizing effect of agents in lower doses.

## 4. Materials and Methods

### 4.1. Culture of Uveal Melanoma Cells Lines

The uveal melanoma cell line UM92.1 and uveal primary melanoma cell lines UMPD2 and UPMM3 were provided by Dr. M. Zeschnigk (Institute of Human Genetics, University Hospital Essen, Essen, Germany) [24,25,26,27,28,29]. The UM cell line Mel270 was provided by Dr. K. Griewank (Department of Dermatology, University Hospital Essen, Germany) [25,26]. All cell lines were authenticated by short tandem repeat profiling according to published data. The cell lines were maintained in an RPMI 1640 medium (UM cell lines) or in a Ham/F12 medium (UPM cell lines) supplemented with 10% fetal calf serum and 1% penicillin-streptomycin (5000 U/mL), respectively. The medium was refreshed two times per week. The cell cultures were incubated in a humified incubator (37 °C, 5% CO_2_).

### 4.2. Cell Viability Assay

The cell cultures were trypsinized and the resultant cell suspension was mixed with an equal amount of 4% trypan blue stain (Merck, Darmstadt, Germany). The number of living and dead cells was determined by trypan exclusion using a TC20 automated cell counter (Bio-Rad, Feldkirchen, Germany).

### 4.3. Tumor Spheroids

The spheroids were generated by seeding 5 × 10^3^ living cells in round bottom 96-well ultra-low attachment plates (Corning, Corning, NY, USA) in 100 µL of the respective cell culture medium. The medium was refreshed two times per week. The spheroid cultures were incubated in a humified incubator (37 °C, 5% CO_2_) for the indicated time of period. Compact spheroids could be generated on day 4 of the culture.

### 4.4. Determination of Spheroid Growth

The spheroids (*n* = 5 each condition) were imaged at day 4, 7 and 12 using a Zeiss Primovert bright-field microscope at 4 × magnification. The images were recorded with a Zeiss Axiocam 105 and ZENcore software and analyzed by using image processing software Fiji ImageJ 1.53c (MPI-CBG, Dresden, Germany). The spheroid size was determined by calculating the cross-sectional area of the spheroids (µm^2^). The spheroid compactness was determined by calculating the optical density of the spheroid area (mgv, mean grey value). In order to determine the living/dead cells, the spheroids (*n* = 8–16 in triplicate) were trypsinized and the resulting cell suspension was subjected to a cell viability assay as described above.

### 4.5. Treatment of Spheroids

The spheroid cultures were irradiated and/or treated with electrochemotherapy (ECT). In terms of combination therapy, the spheroid cultures were first irradiated and additionally treated with ECT within one hour after radiation. As controls, the spheroid cultures remained untreated.

Radiation: spheroid cultures were irradiated in a 100 µL medium in multi-wells with a dose of 5 or 20 Gy by using a ^60^Co source X-ray irradiator (RS320, Xstrahl Ltd., Surrey, UK) at 300 kV, 10 mA and a dose rate of 0.9 Gy/min.

ECT: the spheroids were treated with a 100 µL medium containing 1.0 µg/mL or 2.5 µg/mL bleomycin and electroporated by using two parallel aluminum electrodes 4 mm apart. Eight pulses (100 µs pulse duration, 5 Hz repetition frequency) of a 1000 V/cm or 750 V/cm pulse strength were applied by a voltage pulse generator (Genedrive, IGEA S.p.A., Carpi, Italy). As controls, further samples were treated with bleomycin or electroporation only. After 24 h of treatment, the spheroids were washed with the medium and incubated in a fresh medium for the indicated period of time.

### 4.6. Immunohistochemistry of Tumor Spheroids

The spheroids (*n* = 3–8 each condition) were fixed in buffered formalin (Histofix). Formalin-fixed paraffin embedded sections were cut at a thickness of 3 µm. The Ki67 immunohistochemistry was performed on a Ventana BenchMark Ultra system (Ventana Medical Systems, Tucson, AZ, USA) using clone 30-9, a monoclonal mouse IgG antibody (Cell Signaling Technology, Cambridge, UK). An immunohistochemistry analysis was performed with the following protocol: pretreatment cell conditioning 1, 95 °C, 32 min.; incubation 36 °C, 16 min. The visualization was conducted using an OptiView DAB System (Ventana Medical System). Thereafter, the slides were scanned using a Leica DM4000B microscope, Leica DFC290 camera and analyzed using the Leica Suite version 2.8.1 (Leica Microsystems, Wetzlar, Germany). The slides were reviewed by a board-certified pathologist (ST).

### 4.7. Spheroid Viability Assay

The spheroid cell viability was assessed using the CellTiter-Glo 3D cell viability assay (Promega) according to the manufacturer’s instructions. Equal amounts of CellTiter-Glo and spheroid cultures were mixed by pipetting up and down for 30 s to enable a complete lysis of the spheroid cells and the release of ATP. The mixture was transferred to white opaque-walled multi-well plates (Nunc Micro well). After five minutes of incubation on a shaker (750 rpm) and a further 30 min of incubation, the luminescence was recorded using a reader FluostarOmega (Bmg Labtech, Ortenberg, Germany). The ATP luminescence was given in RLU (relative light units).

### 4.8. Spheroid Cell Survival Assay

Five days after treatment (at day 12), the spheroid cultures (*n* = 12 each condition) were individually transferred to 24-well plate dishes. The spheroids were allowed to attach to the uncoated flat plastic bottom to enable the out-growth of spheroid cells. After eight weeks in the culture, the cell confluence was determined by bright-field microscopy. The adherent cells were fixed with formalin for 5 min and the cell nuclei/DNA were stained with 0.05% Crystal Violet for 30 min. The absorbance of Crystal Violet (CV) was measured at OD 540 nm (au, absorbance units) using a FluostarOmega (Bmg Labtech, Ortenberg, Germany) reader.

### 4.9. Statistical Analysis

A statistical analysis of the data was performed using a two-way ANOVA and Tukey’s multiple comparisons test (GraphPad Prism 8.4.3 software, GraphPad Software Inc., San Diego, CA, USA). A value of *p* < 0.05 was considered statistically significant and significance levels were indicated * *p* < 0.05, ** *p* < 0.01, *** *p* < 0.005, **** *p* < 0.001.

## 5. Conclusions

This study presented the positive radiosensitizing effect and the long-term results of concomitant irradiation and ECT with bleomycin in primary UM 3D spheroids with or without monosomy 3. The results indicated that uveal melanoma cell lines could be radiosensitized after one ECT treatment, making a clinical application practicable. The different levels of radiosensitivity between the various UM cell lines could be attributed to the tumor origin and prior treatments as well as to the intrinsic tumor characteristics and genetics. As shown for the Mel270 and UM92.1 cell lines, the combined treatment with electrochemotherapy and tumor irradiation could be used to treat radioresistant tumors and larger tumor nodules, which would not achieve a satisfactory tumor control with other single-modality treatments. Further studies are necessary for the examination of the time setting and the interval between ECT and irradiation as well as of the effect of repeated ECT sessions and a fractionated irradiation regimen in uveal melanoma tumors.

## Figures and Tables

**Table 1:** Characteristics of uveal melanoma cell lines.

Cell Line	Genetics	Morphology/Doubling Time	References
UM92.1	GNAQ Q209L, Disomy-3, WT BAP1, EIF1AX	Epithelioid/38–58 h	[24,25,26,27]
Mel270	GNAQ Q209P, Disomy-3, WT BAP1	Spindle/43 h	[25,26,28]
UPMD2	GNA11 Q209L, Isodisomy-3, WT BAP1	Epithelioid/150 h	[16,29]
UPMM3	GNAQ Q209P, Monosomy-3, Mutant BAP1	Spindle and epithelioid/100 h	[29]
